# Recognizing Female Reproductive Factors in Interpreting Changes in Disease Burden Among Populations

**DOI:** 10.2188/jea.JE20240123

**Published:** 2024-08-05

**Authors:** Miho Iida

**Affiliations:** Department of Preventive Medicine and Public Health, Keio University School of Medicine, Tokyo, Japan

Reproductive factors in women are closely associated with many health risks. Late menarche decreases mid-life cancer risks, such as endometrial cancer, liver cancer, melanoma, bladder cancer, and cancers of the colon and breast by 2–9%.^1^ Age at menopause is associated with risk of cancers not only specific to women but also common to both sexes, such as lung and thyroid cancers.^2^^,^^3^ Age at menopause may also be applied in the risk stratification of cardiovascular disease in women.^4^ Number of births and breastfeeding are also key factors to consider, particularly in the risk of breast, endometrial, and ovarian cancers. Meta-analyses have shown that for each birth, the risks are reduced by 7–16%, and risks are reduced by 2–4% for every 12 months of breastfeeding.^5^ Compared to women who have had children at a younger age, women who had their first child after age 30 have a higher breast cancer risk.

Women’s reproductive health affects not only their own overall health, but also the health risks of future generations. Breastfeeding provides numerous health benefits to a child and can influence the long-term health trajectories of offspring by supporting growth, immune function, and cognitive development through the antibodies, hormones, and nutrients contained in breast milk. Optimal breastfeeding practices have been associated with lower rates of infectious diseases, obesity, and chronic conditions in children and may confer long-term health benefits into adulthood.^6^ Maternal age at childbearing is associated with distinct health risks for a child. Advanced maternal age is linked to higher rates of pregnancy complications, such as hypertensive disorders, gestational diabetes, preterm delivery, and low birthweight,^7^ which predispose a child to an increased risk of hypertension, chronic kidney disease, and other related noncommunicable diseases later in life.^8^ These are just a few examples, and there are many more studies highlighting the complex relationship between reproductive factors in women and health outcomes of the women themselves and their offspring. These associations underscore the importance of women’s reproductive health, especially its potential long-term impact on overall health of women, as well as the health of future generations.

Identifying trends in health indicators in a population is fundamental to properly assess changes in the burden of disease and to take public health measures. However, changes and trends in reproductive factors over time, and especially their impact on disease burden, have received little attention to date. In this issue, Iwase et al conducted a large-scale study to assess secular changes in reproductive factors by birth year and investigate the trends of age at menarche in more than 60,000 Japanese women born between 1890 and 1991, the longest observation period in the nation.^9^ Using linear and joinpoint regression models, the authors observed not only a significant long-term downward trend in age at menarche over 100 years, but also significant points of change in the trend, specifically 1932, 1946, and 1959, which appeared to coincide with major social and historical events, as well as economic growth in Japan. The authors speculate that social emergencies, such as wars and pandemics, may have affected medical administration and public health, which in turn induced changes in individuals’ economic and nutritional status, leading to changes in their biological factors. Trends of parity, breastfeeding instances, age at first birth, and age at menopause were also investigated among 9,000 to 25,000 women who participated in the study, which also showed change over time. Since individual lifestyles were not investigated in this study, the implications behind the trends were limited. Nevertheless, the study deserves credit for examining the secular trends and changes in age at menarche and other female reproductive factors within a large-scale population for over a century and elucidating factors that drove these shifts using annual Gross Domestic Product per capita data in Japan.

Indeed, social and historical events have influenced human biological events and indirectly influenced disease burden at the population level. For example, liver cancer incidence in Japan has shown its peak in the birth cohort of 1931–1935, which appears to be related to the prevalence of hepatitis C virus infection in this generation, partly due to the use of parenteral amphetamines in the devastated post-World War II society.^10^ Stomach cancer has been steadily declining in Japan, possibly because economic development and improved sanitation have reduced the rate of Helicobacter pylori infection in infancy and childhood in the country.^11^ Another historical event in our country that has influenced disease burden is the suspension of the national human papillomavirus (HPV) vaccination program from 2013–2022. As a result, the burden of cervical disease among women of reproductive age in Japan is expected to increase. Projections through 2028 indicate that if the HPV vaccination and screening rates in Japan remain at the levels during the suspension period, cervical cancer incidence and mortality in the age group of 30 to 69 years will increase, as well as the incidence of cervical intraepithelial neoplasia 3 in the age group of 25 to 49 years.^12^ The approval of low-dose oral contraceptives in 1999 and low-dose pills for the treatment of dysmenorrhea and endometriosis in 2008, as well as the introduction of insurance coverage of infertility treatment in 2022, could also be considered social events that will affect the reproductive factors of future generations of women in Japan and require further attention. As it is already becoming clear that coronavirus disease 2019 has brought a broad range of health effects, trends in reproductive factors need to be tracked, such as decline in fertility as observed in past pandemics.^13^

In conclusion, I hope that this paper by Iwase et al will raise awareness of the significance and importance of obtaining data on reproductive factors over time and that it will be used to interpret changes in disease burden in populations.
